# Postpartum visits in the gynecological emergency room: How can we improve?

**DOI:** 10.1186/s12884-020-02927-7

**Published:** 2020-05-07

**Authors:** Alina Weissmann-Brenner, Ishai Heusler, Renana Manteka, Mordechai Dulitzky, Micha Baum

**Affiliations:** 1 Chaim Sheba Medical Center, Tel HaShomer Hospital, Ramat Gan, Israel; 2 Faculty of Medicine, Tel Aviv University, Tel Aviv, Israel

**Keywords:** Postpartum, Gynecological emergency room, Cesarean birth, Hypertensive disorders

## Abstract

**Background:**

The attendance to the gynecological-emergency-room (GER) of women only a few weeks following previous discharge after birth comprises a medical as well as social problem. The objective of the study was to characterize the postpartum women that attended the GER, and depict the leading etiologies and risk-factors that lead them to attend the GER, and to examine correlations between their medical findings at discharge and the reasons for their attendance to the hospital.

**Methods:**

All women that attended the GER between 01/01/2016 and 30/09/2016 during 6 weeks after birth were included. The variables assessed were: medical history, mode of birth, maternal complications of birth, diagnosis at the GER, treatment received and readmission.

**Results:**

There were 446 visits of 413 women (5.6% of all deliveries). Most were generally healthy after their first normal vaginal birth with no complications during or following birth. 38.7% had a cesarean birth (*p* < 0.001). The most common causes of the visits were fever (30.3%), problems in episiotomy or surgical scar (26.6%) and abdominal pain (25.7%). Women with hypertensive disorders during birth had significantly more hypertensive problems in the GER. Diabetic women suffered statistically more from hypertensive disorder in the GER. 33.2% were examined and found healthy. Seventy-two women (1% of all deliveries) were hospitalized, most of them due to infection. Only 7.5% were referred to the GER due to bleeding.

**Conclusions:**

Postpartum women are at risk of morbidities, especially following cesarean sections and in women with hypertensive disorders of during pregnancy. Scheduled visits to high-risk women to attend outpatient clinic sooner are recommended.

## Background

The postpartum period, defined as the first 42 days following birth, is characterized by many maternal physiological as well as emotional and social changes. Many mothers have insufficient knowledge about adequate postpartum care, the possible complications and the preventive measures or treatment modalities in cases of illnesses [1, 2].

Several studies focused on parenting support in the postpartum period, and emphasized the importance of continuity of treatment after discharge following delivery [1, 2]. However, there is insufficient data regarding maternal care during this vulnerable period. A wide range of physiological changes that occur in the postpartum period may mask illnesses. For example, fatigue, anxiety, stress, depression and sleep disorders may mask anemia, infection or hypertension; vaginal bleeding may be normal but may also result from retained placenta; and urinary incontinence may be a sign of urinary tract infection [3–5].

The attendance of puerperal women to the gynecological emergency room (GER) only a few days or weeks following previous discharge after birth, comprises a medical as well as social problem. One study found that 27% of women who had complicated pregnancies attended the GER post discharge following delivery. However, data regarding the types of complications that lead to women attending the GER is lacking [6]. The readmission rate is reported to be 1–2.16%, with an increase over the years [7–13].

The objective of the study was to characterize the postpartum women that attended the GER, to describe the causes and medical diagnosis of their visits, treatment and hospitalization. Their previous hospitalization was analyzed with special emphasis on medical problems such as diabetes and hypertensive disorders during pregnancy and infections and bleeding during delivery as well delivery by cesarean section. Our goal was to identify women at increased risk for postpartum complications, and to improve the treatment, postpartum care and follow up of these vulnerable women.

## Methods

The study population included all the women that attended the GER of the Chaim Sheba Medical Center between 01/01/2016 and 30/09/2016 during the 6 weeks following birth. The study was approved by the institutional review board (IRB) of the Chaim Sheba Medical center. Due to the retrospective nature of the study this IRB approved not to obtain informed consent.

Postpartum was defined as the first 6 weeks following birth as accepted in previous publications [12].

Inclusion criteria were women who delivered at the Chaim Sheba medical center that attended the GER in the first 6 weeks following birth.

Data was derived from the department’s women database. Women’s medical history, pregnancy outcomes, and medical data at admission to the GER are routinely entered into the hospital’s database.

The variables assessed in the study were: maternal age, parity, medical history, mode of birth, maternal complications at birth, fever, examination before discharge following birth, reasons and diagnosis of the GER visits, treatment received at GER and hospitalization.

The definition of maternal infectious condition included fever accompanied by clinical signs and symptoms that resulted in antibiotics treatment during or following birth.

Hypertensive disorders included hypertensive disease as a background medical condition, and any pregnancy related hypertensive disorder (including pregnancy induced hypertension, preeclampsia and eclampsia), treatment with antihypertensive medications, and postpartum hypertensive disorder.

### Statistical analysis

The method of analysis in this article is largely descriptive, presenting the study variables as percentages for dichotomous and categorical characteristics. Mean, standard deviation, median and range were calculated for continuous variables such as age and length of stay. Causes of visit to the hospital, medical diagnosis, treatment, hospitalization and length of stay were compared between women with and without infections, bleeding hypertensive disorders, diabetes during birth and birth by cesarean section. Distribution of complications following birth, and causes of visit to GER in women with subsequent normal and abnormal examination, were presented. Differences of the above mentioned variables between the groups were examined by Chi-squared tests in the case of categorical and dichotomous variables or when relevant, by Fisher’s exact test. Unpaired T-test was used for the comparison of length of stay between the study groups. A value of *p* < 0.05 was considered statistically significant.

## Results

During the study period there were 7371 deliveries at the Chaim Sheba Medical center, 5502 (74.6%) were normal vaginal deliveries, 1869 (25.3%) cesarean sections and 412 (5.6%) operative vaginal deliveries.

In total, 413 puerperal women attended the GER. Of them, 389 visited the GER once, 18 visited the GER twice, three women came three times to the GER and three women came four times to the GER at the first 6 weeks following their childbirth. Overall there were 446 visits to the GER by 413 women (5.6% of all deliveries).

The characteristics of the study population are presented in Table 1. Most of them were generally healthy after their first normal vaginal birth with no complications following birth. A high rate of 16.7% had diabetes. The characteristics of women’ birth are presented in Table 2. Significantly more women delivered by a cesarean section (38.2%), compared to the 25.3% in the entire population that delivered by cesarean section during the study period in our medical center. The 26 women (6.3%) that had medical findings during their discharge from the hospital, had improving hematoma of cesarean scar, headache, improving leg edema, improving abnormal liver or renal functions in laboratory tests. Ten women out of the 26 that were discharged with medical findings (38.5%) were readmitted to the hospital.

**Table 1:** Distribution of demographic and selected clinical characteristics of the study population

	No.	%
	413	100
**Age**		
Average	32.9 ± 5.4	
Median (range)	(19–54) 32	
**40>**	361	87.4
**40≤**	52	12.6
**Gravidity**		
Average	2.34 ± 1.73	
Median (range)	(1–12)2	
**Parity**		
1	232	56.2
2–4	170	41.2
5–11	11	2.7
**Medical history**		
Generally healthy	247	59.8
Endocrine disease	90	21.8
Neurological disease	27	6.5
Pulmonary disease	19	4.6
Gastrointestinal disease	17	4.2
Gynecological disorder	17	4.2
Mental disorder	16	3.9
Hypertensive disorder	13	3.1
Rheumatological disease	8	1.9
Morbid obesity	5	1.2
Urinary disease	5	1.2
Oncological disease	5	1.2
Cardiac disease	4	1.0
Orthopedic problem	4	1.0
Infectious disease	3	0.7
Genetic disease	2	0.5
**Diabetes**		
No	344	83.3
Yes	69	16.7
GDMA1	42	10.2
GDMA2	24	5.8
PGD	3	0.7

*GDM* gestational diabetes mellitus

*PGD* pre-gestational diabetes

**Table 2:** Distribution of delivery related characteristics of the study population

	No.	%
**Mode of delivery**		
NVD	222	53.8
Vacuum	27	6.5
Forceps	4	1.0
Cesarean section	160	38.7
**Complications during delivery**		
No	387	93.7
Fever	8	1.9
Bleeding	19	4.6
**Episiotomy**		
No	313	75.8
Yes	100	24.2
**Perineal tear**		
No	291	70.5
Grade 1–2	117	28.3
Grade 2–4	4	1.0
Cervical tear	1	0.2
**Obstetrical maneuvers**		
No	395	95.6
Manual revision of uterine cavity	13	3.1
Lysis of placenta	5	1.2
**Complications following delivery**		
No	335	81.0
Yes	78	18.9
Infection	9	2.2
Hypertension	8	1.9
Bleeding	32	7.5
Urinary	12	2.9
Placental residua	9	2.2
SVT	6	1.5
Complications of anesthesia	4	1.0
Allergy	1	0.2
Psychosis	1	0.2
**Medical findings during discharge**		
No	387	93.7
Yes	26	6.3
**Discharge with antibiotics**	32	7.7
**Early discharge due to maternal request**	5	1.2
**Length of hospitalization stay (days)**		
Mean ± SD	4.0 ± 3.3	
Median (range)	3.0 (1–37)	

*NVD* normal vaginal delivery

*SVT* superficial vein thrombosis

The reason for the GER visit, the medical diagnosis and the treatment are presented in Table 3. The most common causes were fever (30.3%), problems in episiotomy or surgical scar (26.6%) and abdominal pain (25.7%). A third (34.7%) were examined and found healthy, 56.4% needed medical treatment, and 72 women comprising 1% of all deliveries were hospitalized (comprising 16% of all visits to the GER and 17.4% of all the women), most of them due to infection.

**Table 3:** Distribution of cause of referral to GER^a^, medical diagnosis and treatment in the GER

	No.	%
**Cause of referral to GER**		
Fever	125	30.3
Abdominal pain	106	25.7
Bleeding	62	15.0
Problem in scar	48	11.4
Problem in episiotomy/tear	110	26.6
Neurological problems	68	16.5
Orthopedic problems	28	6.8
Hypertension	17	4.1
Gastrointestinal problem	11	2.7
Venous thromboembolism	8	1.9
Dermatological problem	7	1.7
Shortness of breath	6	1.5
Cardiac problem	6	1.5
Hemorrhoids	3	0.7
Urinary problem	2	0.5
Mental problem	2	0.5
**Medical diagnosis in GER**		
Normal examination	143	34.7
Infection	103	24.9
Bleeding	24	5.8
Hypertension	15	3.6
Problem in scar/episiotomy/tear	63	15.3
Abdominal pain	29	7.0
Neurological problem	16	3.9
Placental residua	8	1.9
Urinary problem	7	1.7
Venous thromboembolism	4	1.0
Other^b^	14	3.4
**Need for medical consultants in GER**		
**No**	330	79.9
**Yes**	83	20.1
**Treatment in GER**		
No need of treatment	180	43.6
Medical treatment	182	44.1
Removal of stich	46	11.1
Treatment of scar	24	5.8
Invasive/operative treatment	3	0.7
Insertion/removal of catheter	3	0.7
Other	3	0.7
**Medical treatment in GER**		
No need	231	55.9
Antibiotics	103	24.9
Analgesia	58	14
Antihypertensive medications	12	2.9
Uterotonic medications	13	3.1
Treatment of hemorrhoids	5	1.2
Antipsychotic medications	2	0.5
Blood products	1	0.2
Other	20	4.8
**Surgical treatment**		
**No**	410	99.3
**Yes**	3	0.7
Blood Patch	1	0.2
Angiography	1	0.2
Dilatation and curettage	1	0.2
**Self discharge from GER**		
No	398	96.4
Yes	15	3.6
**Hospitalization following GER visit**		
No	341	82.6
Yes	72	17.4
**Causes of hospitalization**		
Infection	43	59.7
Bleeding	9	12.5
Problems in scar/episiotomy /tear	5	6.9
Hypertension	8	11.1
Neurological problems	8	11.1
Venous thromboembolism	2	2.8
Placental residua	2	2.8
Mental problems	1	1.4
Abdominal pain	1	1.4
Ascites	1	1.4
**Department of hospitalization**		
Gynecology	69	97.3
Surgery	2	2.8
Neurology	1	1.4

^a^GER Gynecological Emergency Room

^b^Other: ascites, dermatological problems, gastrointestinal problems, mental problems

We compared women who had abnormal examination at discharge following birth to women with normal examination, Table 4, and found no differences in the number of women who had complications following birth.

**Table 4:** Distribution of complications during delivery and causes of referral to GER in patients with subsequent normal and abnormal examination in the GER

	Normal examination		Abnormal examination		P
	No.	%	No.	%	
	143	100	270	100	
**Complications following delivery**					
No complications	113	79	222	82.2	0.6
With complications	24	16.7	54	20	
Infections	4	2.8	4	1.5	
Hypertensive disorders	3	2.1	5	1.8	
Bleeding	7	4.9	23	8.5	
Placental residua	3	2.1	6	2.2	
Superficial vein thrombosis	2	1.4	2	0.7	
Urinary problems	4	2.8	8	3	
Other^a^	1	0.7	7	2.6	
**No. of complications**					
1	21	14.7	51	18.9	0.4
2	3	2.1	2	0.7	
3	0		1	0.4	
**Cause of referral to GER**					
Infection	25	17.5	100	37	< 0.001
Bleeding	27	18.9	35	13	0.06
Hypertensive disorder	3	2.1	14	5.2	0.13
Problem in episiotomy	48	33.6	62	23	0.006
Problem in scar	17	11.9	31	11.5	0.7
Abdominal pain	25	17.5	81	30	0.015
Neurological problem	20	14	48	17.8	0.5
Orthopedic problem	7	4.9	21	7.8	0.3
Gastrointestinal problems	6	4.2	5	1.8	0.12
Venous thromboembolism	3	2.1	5	1.8	0.5
Other	9	6.3	17	6.3	0.9

^a^Other: Anesthesia, abdominal pain, allergy and psychosis Mental, urinary, cardiac, dermatological problems, shortness of breath, hemorrhoids

Characterization of women whose birth was complicated with infection, bleeding or hypertensive disorders is presented in Table 5. Significantly more women with hypertensive disorders during pregnancy visited the GER and were readmitted to the hospital (*p* < 0.001).

**Table 5:** Causes of referral to the hospital, medical diagnosis, treatment and hospitalization in patients by presence of infections, bleeding and hypertensive disorders during delivery

	Hypertensive disorders					Bleeding					Infections				
	Yes		No		P	Yes		No		P	Yes		No		P
	%	n	%	n		%	n	%	n		%	n	%	n	
Total	26	100	387	100		51	100	362	100		38	100	375	100	
**Cause of referral to GER**															
Infection	5	19.2	120	31.0	0.15	16	31.4	109	30.1	0.8	15	39.5	110	29.3	0.2
Bleeding	0		62	16.0	0.01	8	15.7	54	14.9	0.9	7	18.4	55	14.7	0.5
Hypertension	13	50.0	4	1.0	0.001	4	7.8	13	3.6	0.15	0		17	4.5	0.2
Abdominal pain	7	26.9	99	25.6	0.5	15	29.4	91	25.1	0.5	9	23.7	97	25.9	0.8
Problem in scar	2	7.7	46	11.9	0.4	2	3.9	46	12.7	0.04	6	15.8	42	11.2	0.4
Problem in episiotomy	3	11.5	107	27.6	0.05	7	13.7	103	28.5	0.03	11	28.9	99	26.4	0.7
Shortness of breath	2	7.7	4	1.0	0.05	1	2.0	5	1.4	0.5	1	2.6	5	1.3	0.4
Neurological problem	11	42.3	57	14.7	0.001	12	23.5	56	15.5	0.15	5	13.2	63	16.8	0.4
Other^a^	1	3.8	57	14.7	0.2	4	7.8	54	14.9	0.2	6	15.8	52	13.9	0.8
**Diagnosis in GER**															
Normal exam	4	15.4	133	34.4	0.03	16	31.4	123	34.0	0.4	18	47.4	119	31.7	0.05
Infection	4	15.4	99	25.6	0.18	16	31.4	87	24.0	0.3	11	28.9	92	24.5	0.5
Bleeding	2	7.7	22	5.7	0.5	5	9.8	19	5.2	0.16	2	5.3	22	5.9	0.6
Hypertension	15	57.7	0		< 0.001	2	3.9	13	3.6	0.6	0		15	4.0	0.2
Problem in scar/episiotomy	1	3.8	62	16.0	0.07	4	7.8	59	16.3	0.08	4	10.5	59	15.7	0.3
Other^a^	1	3.8	54	13.9	0.2	10	19.6	45	12.4	0.2	3	7.9	52	13.9	0.4
**Treatment in GER**															
Yes	16	61.5	218	56.3	0.6	30	58.8	204	56.4	0.7	16	42.1	218	58.1	0.057
No	10	38.5	169	43.7		21	41.2	158	43.6		22	57.9	157	41.9	
**Hospitalization**															
Yes	10	38.5	62	16.0	0.004	13	25.5	59	16.3	0.08	5	13.2	67	17.9	0.3
No	16	61.5	325	84.0		38	74.5	303	83.7		33	86.8	308	82.1	

^a^Other: orthopedic, cardiac, urinary, gastrointestinal problems, hemorrhoids, venous thromboembolism

**Fig. 1:**
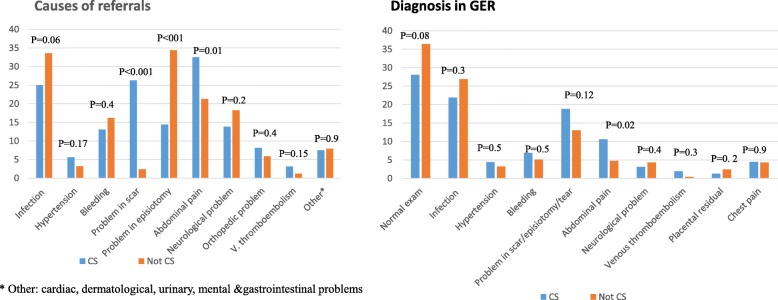
Causes of referrals to the GER and diagnoses in patients with cesarean delivery

**Fig. 2:**
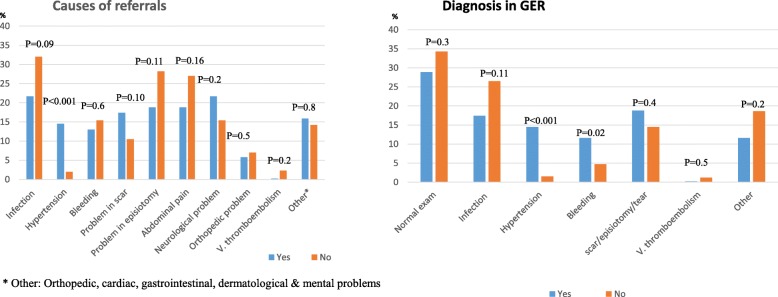
Causes of referrals to the GER and diagnoses in patients with diabetes mellitus

Comparison of reasons for GER visits, treatment and hospitalization in women following cesarean birth and in women with diabetes are presented in Figs. 1 and 2, respectively. The statistically significant causes of their GER visits were problems in the cesarean scar or episiotomy and of abdominal pain. No increased rate of bleeding or infection was found in these women.

Significantly more women with diabetic complications utilized the GER due to hypertensive disorders, and needed treatment in the GER (*p* < 0.001).

## Discussion

The present study demonstrated that 5.6% of all postpartum women visited the GER during the 6 weeks following birth. The most common causes of the GER visits were fever, abdominal pain and problems in episiotomy or surgical scar, and to a lesser extent vaginal bleeding. While hypertension and its complication were more common in women with known hypertensive disorders during pregnancy, most women were discharged without any complaints or clinical findings on examination.

Infection may be considered a major clinical risk in the postpartum women. A third of the women in the present study were diagnosed with an infectious condition in the GER. Similarly, Belfort et al., in their study of postpartum readmissions, reported that most of the reasons for readmission were infections [11]. Yokoe et al. described that 94% of postpartum infections became manifest only after the discharge from the hospital discharge [13]. We found more visits to the GER due to infections in women previously treated for infectious conditions immediately following childbirth, but this was not statistically significant. Caregivers may not reinforce women’s intake of medications following discharge. Women may stop medications such as antibiotics if they feel well, or they may not take the prescribed dose, therefore causing relapse of infection. Furthermore, previous studies suggested that the immediate postpartum period may be a time of generalized immune suppression, thereby placing these women at increased risk of infection [12, 14–17]. Increased attention and explanation of the importance of the intake of medications should be given to the women prior to their discharge.

A similar challenge lies within women with hypertensive disorders. The present study demonstrated a statistically significant higher rate of hypertensive disorders in the women who attended the GER and suffered from hypertension during pregnancy or following birth. Previous studies report that women with hypertension had a higher proportion of acute care visits and higher rates of hospitalization in the postpartum period, including 11–44% rates of postpartum eclampsia [18, 19]. Some women are discharged with antihypertensive medications. Here too we are limited in the verification of the women’ compliance to medications. Health professionals cannot monitor the actual intake of anti-hypertensive drugs outside the hospital. Most of the postpartum women with hypertension in the GER in the present study needed treatment and a high rate of 38.5% were readmitted. More attention should be given to the health education of hypertensive women before discharge regarding intake of medications and early identifications of hazardous signs of preeclampsia.

Similar to previous studies, the present study demonstrated that the mode of birth also poses a risk for postpartum visits in the GER, with statistically more women who delivered by a cesarean section visited the GER. The statistically significant reasons for their visits to the GER were abdominal pain, problems in the scar and problems in the episiotomy. The 21.9% rate of infection in women following cesarean section found in our study is similar to previous studies [10, 20–22].

We found a significantly higher rate of 16.7% of women with diabetes mellitus that visited the GER compared to the rate of 6–7% of diabetes in the general population [23]. Most of them suffered from hypertensive disorders and needed treatment in the GER. Similarly, Harris et al. and Clapp et al. reported more visits to the GER and more hospital readmissions in women whose pregnancies were complicated with diabetes mellitus [6] [9].

Medical problems necessitating visits to the GER pose a great burden on the women herself, her close environment and the medical system. Visit to the emergency room costs approximately 230 Ero (257$). Visit to the outpatient clinic costs approximately 38Ero (43$), making the visit to an outpatient clinic more cost effective. An earlier visit to an outpatient clinic should be recommended in high risk women with hypertensive disorders, infections, diabetes and following cesarean sections.

We acknowledge several limitations in our study: We did not analyze information regarding who referred the women to the GER. Therefore we do not have information on potential treatment in outpatient clinics. Referrals to outpatient clinics may decrease the GER visits. However, our study demonstrated a similar rate of GER visits as previously reported by Clark et al. and Brown et al. (4.8 and 7.8%, respectively [3, 13]). Furthermore, in a recent study Vikstrom al demonstrated that although women were scheduled planned follow-up visits during the first week following birth, 50% of the GER visits were unplanned [22].

## Conclusions

Fever, abdominal pain, problems in episiotomy or surgical scar, and vaginal bleeding were the most common causes of the GER visits found in the present study. Postpartum women following cesarean sections and women with hypertensive disorders during pregnancy are at increased risk of comorbidities in the postpartum period. We recommend meticulous explanations to the women both in the hospital before discharge and in the outpatient clinics regarding the postpartum care in both vaginal and cesarean deliveries. Special emphasis should be made on the adherence to medical treatment received at home, in order to avoid physical deterioration. Scheduled visits to high-risk women to attend outpatient clinic sooner are recommended.

## Data Availability

The datasets used and/or analysed during the current study are available from the corresponding author on reasonable request.

## Abbreviation

GER: Gynecological-emergency-room
